# UV-Assisted 3D Printing of Glass and Carbon Fiber-Reinforced Dual-Cure Polymer Composites

**DOI:** 10.3390/ma9070583

**Published:** 2016-07-16

**Authors:** Marta Invernizzi, Gabriele Natale, Marinella Levi, Stefano Turri, Gianmarco Griffini

**Affiliations:** Department of Chemistry, Materials and Chemical Engineering “Giulio Natta”, Politecnico di Milano, Piazza Leonardo da Vinci 32, Milano 20133, Italy; marta.invernizzi@polimi.it (M.I.); gabriele.natale@polimi.it (G.N.); marinella.levi@polimi.it (M.L.); stefano.turri@polimi.it (S.T.)

**Keywords:** 3D printing, composites, carbon fiber, glass fiber, interpenetrating polymer networks, dual cure, UV curing

## Abstract

Glass (GFR) and carbon fiber-reinforced (CFR) dual-cure polymer composites fabricated by UV-assisted three-dimensional (UV-3D) printing are presented. The resin material combines an acrylic-based photocurable resin with a low temperature (140 °C) thermally-curable resin system based on bisphenol A diglycidyl ether as base component, an aliphatic anhydride (hexahydro-4-methylphthalic anhydride) as hardener and (2,4,6,-tris(dimethylaminomethyl)phenol) as catalyst. A thorough rheological characterization of these formulations allowed us to define their 3D printability window. UV-3D printed macrostructures were successfully demonstrated, giving a clear indication of their potential use in real-life structural applications. Differential scanning calorimetry and dynamic mechanical analysis highlighted the good thermal stability and mechanical properties of the printed parts. In addition, uniaxial tensile tests were used to assess the fiber reinforcing effect on the UV-3D printed objects. Finally, an initial study was conducted on the use of a sizing treatment on carbon fibers to improve the fiber/matrix interfacial adhesion, giving preliminary indications on the potential of this approach to improve the mechanical properties of the 3D printed CFR components.

## 1. Introduction

Three-dimensional (3D) printing technologies are nowadays widely employed in several different engineering fields (mechanical, biomedical, aerospace, electronics and more). Indeed, the possibility to create and modify a digital model and rapidly convert it into a physical object by means of 3D printing has opened many opportunities in terms of product customization, process cost reduction and design versatility [1]. These characteristics have allowed 3D printed structures to be directly employed in a variety of contexts as microsensors and actuators, fuel nozzles, scaffolds for cellular growth, home furniture objects, jewels and footwear, to name but a few. Such a large range of accessible applications can be correlated with the large number of currently available 3D printing technologies that allow us to process a constantly increasing number of materials, including polymers, metals, powders, fibers, ceramics, clay and food [2,3].

Among the most widely employed additive manufacturing technologies in the past few years, fused deposition modeling (FDM) has been demonstrated to be a very cost-effective and versatile approach to produce 3D objects of arbitrary shapes in a relatively straightforward fashion, easily accessible also by non-specialized personnel. FDM 3D printers are typically fed with polymeric thermoplastic filaments which are heated above their softening (melting) temperature when passing through an extruding nozzle, whose motion is defined by a computer-controlled three-axis positioning system. While exiting the nozzle, the softened plastic filament cools down, thus rapidly hardening and forming the 3D printed object via a layer-by-layer deposition approach [4,5]. One intrinsic limitation of FDM is the heating step during the extrusion process, which clearly prevents the use of thermosetting polymeric systems as feed. To partially circumvent this issue, other low-cost 3D printing technologies have been very recently demonstrated in the literature, including the so-called liquid deposition modeling (LDM) [6,7]. This approach allows us to extend the range of accessible polymeric materials also to thermosetting systems, as liquid resins with appropriate rheological profiles can be directly processed through a syringe dispenser, followed by post-treatments that allow for the curing reaction to occur and the solid 3D object to form.

An interesting evolution of the LDM approach is represented by UV-assisted 3D (UV-3D) printing, in which the feed material is constituted by photocrosslinkable resin systems that can be cured at room temperature right after exiting the extrusion nozzle by means of UV-light irradiation [8]. While several examples of successful fabrication of 3D objects by means of UV-3D printing have appeared in the literature in the past few years [9,10,11,12,13,14,15], most of these works have focused on the realization of functional 3D structures such as free-standing or spanning micro-features, in some cases incorporating conductive nanofillers to directly embed electrical functionalities in the printed component. On the other hand, very few demonstrations of the use of this technique for the fabrication of 3D structural parts have been proposed, mainly due to the fundamentally limited mechanical properties of the resin systems typically employed in UV-3D printing (in most cases acrylates) which do not allow for the formation of objects with adequate mechanical response [8]. 

As a way to partially overcome these limitations, a carbon-fiber reinforced (CFR) dual-cure thermosetting polymeric system was recently proposed based on a sequential interpenetrating polymer network (IPN) for UV-3D printing applications [16]. The simultaneous presence of a photocurable (acrylate-based) and a thermocurable (epoxy-dicyanediamide based) component in the feed formulation allowed to achieve fast curing kinetics of this ink during the 3D printing process (thanks to the presence of the acrylate-based photocurable resin) while concurrently ensuring the formation of a fully crosslinked 3D structure with excellent mechanical properties upon subsequent high-temperature (200 °C) thermal curing of the epoxy-based resin. 

In this work, the push to lowering the processing temperature and to using safer chemicals has led us to the development of new dual-cure interpenetrating polymer networks based on photocurable acrylics and thermocurable anhydride-epoxy systems. Due to the chemical nature of this different hardening chemistry, thermal-curing could be performed at significantly lower processing temperatures (140 °C) compared to previous reports, while prospecting mechanical properties comparable with conventional amide-based systems [17,18,19,20,21]. By incorporating reinforcing fibers into this novel dual-cure polymeric matrix, CFR and glass-fiber reinforced (GFR) composite formulations could be obtained. A thorough rheological characterization of these dual-cure formulations allowed to define their UV-3D printability window. Subsequently, UV-3D printed macrostructures based on these GFR and CFR composite formulations were successfully demonstrated, giving a clear indication of their potential use in real-life structural applications. Differential scanning calorimetry (DSC) and dynamic mechanical analysis (DMA) highlighted the good thermal stability and mechanical properties of the printed parts. In addition, uniaxial tensile tests were used to assess the reinforcing effect of the fibers on the UV-3D printed objects. Finally, an initial study was also conducted on the use of a sizing treatment on carbon fibers to improve the fiber/matrix interfacial adhesion [22,23,24], which gave preliminary indications on the potential of this approach to improve the mechanical properties of the corresponding 3D printed CFR components.

## 2. Results and Discussion

### 2.1. Rheological Characterization of the Composite Inks

**Figure 1:**
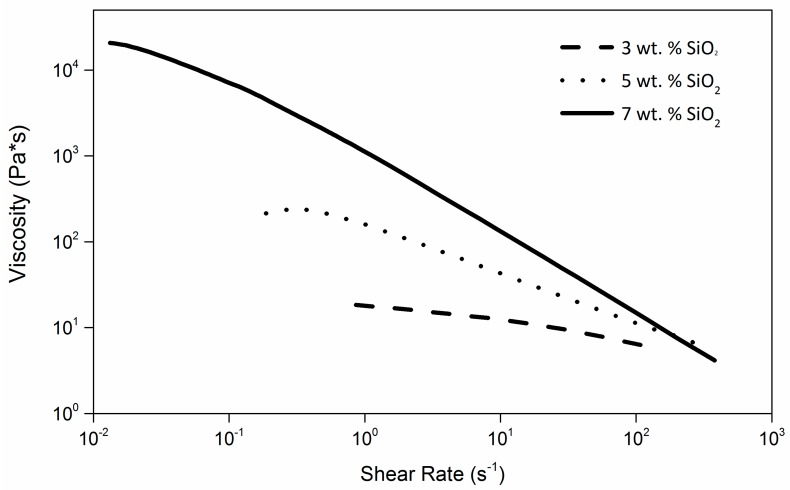
Viscosity curves of B33 formulations (dual-cure blend containing 33 wt. % of photocurable acrylic component) at increasing SiO_2_ content.

The 3D printability of liquid resins via LDM technology is largely dependent on their rheological behavior, which needs to be carefully tuned to allow the production of well-defined printed features. To this end, a preliminary rheological study was performed on the starting dual-cure blend containing 33 wt. % of acrylic photocurable component (B33) by adding increasing amounts of fumed silica as thixotropic agent. As shown in Figure 1, where the viscosity curves of B33 at increasing silica concentration are presented, a clear shear thinning response is observed on all formulations. This effect is found to be more pronounced at higher SiO_2_ concentrations, thus confirming the effective shear thinning abilities of this inorganic filler on the dual-cure blend considered here. 

By fitting the viscosity curves with a power-law fluid relationship described by the following equation:

(1) $$\eta =K\gamma^{\left(n-1\right)}$$

in which *K* and *n* are the consistency index and the power-law index, respectively, the rheological response of the different formulations could be evaluated (see Figure S1 and Table S1 in the Supplementary Materials for calculations). In particular, a correlation could be drawn between the calculated *K* values and the practical printability of each formulation at varying SiO_2_ concentrations. To do so, actual printing tests were carried out on these blends and the integrity of the extruded wet filament after exiting the extrusion nozzle and being deposited on the printing plate was visually evaluated and used as probe of the actual printability of the liquid material. Within the operating limits of the 3D printing apparatus used here, it was found that only SiO_2_-doped B33 formulations characterized by *K* values higher than 790 Pa·s^n^ enabled satisfactory 3D printing behavior, which is in line with recent reports on analogous systems [8]. As a result, 7 wt. % was selected as the minimum SiO_2_ concentration to be used in the starting B33 formulation in all successive characterizations and 3D printing tests.

**Figure 2:**
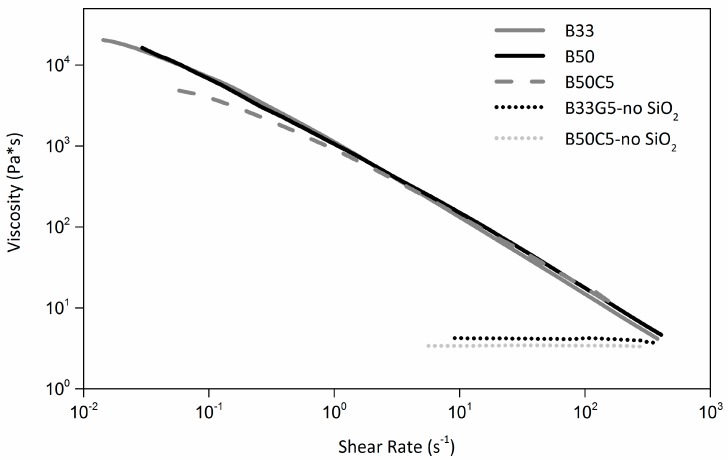
Viscosity curves of SiO_2_-containing B33, B50 and carbon fiber-reinforced (CFR) (B50C5) composite formulations. For comparison, CFR and glass fiber-reinforced (GFR) composite formulations without the addition of SiO_2_ are also presented (B50C5-noSiO_2_ and B33G5-noSiO_2_, respectively).

Ascertained the need of a minimum (7 wt. %) amount of SiO_2_ to allow optimal printability of the starting B33 resin, formulations of dual-cure blends incorporating short glass or carbon fibers were investigated. In particular, a fixed amount of reinforcing filler was added to the starting dual-cure blend, namely 5 wt. % both in the case of glass-fiber reinforced (GFR) and carbon-fiber reinforced (CFR) composite precursor formulations. In view of the use of these resin systems for UV-3D printing applications, efficient interaction between the polymer matrix and the UV light source must be ensured. When CFR composites are considered, it was recently shown that a higher fraction of photocurable resin is typically required to allow appropriate processing [16]. This is mainly due to the presence of the black carbon fibers that partially absorb the incident UV light and reduce to some extent the crosslinking efficiency of the photocurable resin component. To this end, a preliminary assessment of the effect of acrylic photocurable resin concentration (increased to 50 wt. %, leading to the B50 blend) on the rheological profile of the SiO_2_-filled (7 wt. %) starting dual-cure formulation was carried out. As shown in Figure 2, no substantial differences were observed between B33 and B50 over a broad shear-rate range (0.05–500 s^−1^), clearly indicating a negligible effect of the amount of photocurable resin on the rheological properties of the starting dual-cure formulation. Accordingly, B50 blend was employed as polymer matrix for CFR composites while GFR systems were based on B33 blend. The rheological profiles of the resulting formulations (from here on referred to as B50C5 and B33G5 for CFR and GFR composites, respectively) are presented in Figure 2, together with the viscosity curves of the same systems without the addition of SiO_2_ (B50C5-noSiO_2_ and B33G5-noSiO_2_). As expected, the simple addition of carbon or glass fibers to the dual cure blend with no SiO_2_ does not allow us to achieve desirable rheological properties, as both systems exhibit a clear Newtonian behavior. Therefore, also in the case of polymer composite formulations, the addition of a thixotropic agent is necessary for optimal 3D printability. Accordingly, a clear shear thinning behavior is observed on SiO_2_-filled fiber-reinforced composite formulations (a representative curve for a fiber-reinforced formulation (B50C5) is presented in Figure 2).

### 2.2. Dynamic Mechanical Analysis (DMA)

Results of DMA measurements carried out on selected dual-cured IPN resins and composites are presented in Table 1. During the 3D printing fabrication process, the specimens were exposed to UV light for 15 min.

As evident from Table 1, the amount of photocurable resin in the blend formulation does not appear to influence significantly the mechanical response of the resulting unfilled crosslinked samples in the small deformation region. Indeed, a storage modulus of 3.15 GPa is found for both B33 and B50 systems, likely indicating that the mechanical properties of the unfilled 3D printed materials are mainly reflecting the response of the thermocurable component of the blend at these concentrations. Consistent trends are also observed on the *T*_g_ of these materials as inferred from the temperature at the tan*δ* peak in DMA curves, which is found to be independent on the amount of photocurable resin in the dual-cure blend. On the other hand, the addition of reinforcing fibers is found to increase significantly the mechanical response of the dual-cure polymer matrix, irrespective of the type of filler employed. In particular, a 36% and 64% enhancement of the storage modulus is observed in GFR (from 3.15 to 4.30 GPa) and CFR (from 3.15 to 5.18 GPa) composite samples, respectively. These trends suggest that a homogeneous filler dispersion in the polymer matrix could be achieved, thus resulting in efficient interaction between the reinforcing fibers and the polymer and in turn in improved mechanical properties. Compared to the corresponding unfilled dual-cure blends, the *T*_g_ of GFR composite (B33G5) is found to increase (155 °C) while no substantial *T*_g_ modifications are observed on the CFR composite (B50C5). This latter behavior may be explained by considering that upon UV-exposure occurring during the UV-3D printing process of this formulation, carbon fibers may partially shield the photocurable resin and prevent it from fully interacting with UV photons. This may lead to a lower degree of crosslinking of the photocurable component that may in turn yield a lower-than-expected *T*_g_ value. An analogous behavior was recently reported on similar systems [16].

### 2.3. Differential Scanning Calorimetry (DSC)

To further investigate the thermal transitions occurring in the as-prepared unfilled and reinforced dual-cure materials, DSC analyses were carried and the results are also reported in Table 1. The observed trends on unfilled materials are comparable to those found by DMA, where the *T*_g_ is shown to be independent of the amount of photocurable component in the dual-cure blend. In addition, consistent trends are also observed on the CFR polymer composite material that exhibits a slightly lower *T*_g_ value compared to the corresponding unfilled material, likely due to uncomplete crosslinking of the photocurable component of the blend, as also observed during DMA tests. It is interesting to note that for both unfilled and reinforced systems, one single *T*_g_ is observed both in DSC and DMA measurements, thus demonstrating the microstructural homogeneity of the as prepared IPN materials.

The Halpin-Tsai model for random and discontinuous fiber-reinforced polymers was used to correlate the experimental data with theoretical prediction of the mechanical properties of the composite systems investigated here, based on the following equations [25]:

(2) $$E=E_{m}\frac{1+\eta \zeta \nu_{f}}{1-\eta \nu_{f}}$$

(3) $$\eta =\frac{E_{f}/E_{m}-1}{E_{f}/E_{m}+\zeta }$$

with *E*, *E_m_*, *ζ*, *v_f_* and *E_f_* being the modulus of the composite material, the modulus of the matrix, the fiber aspect ratio, the volume fraction of the fibers and the modulus of the fiber, respectively.

It is interesting to note that an excellent match between the measured moduli and the limiting theoretical values extrapolated from the Halpin-Tsai model was achieved for both GFR and CFR polymer composite systems, with calculated values of 4.65 GPa and 5.58 GPa for B33G5 and B50C5, respectively (Table 1). This is a further demonstration of the high level of dispersion of the fillers into the polymer matrices, which results in optimized mechanical response of the reinforced systems.

### 2.4. Uniaxial Tensile Tests

Following the preliminary indications obtained from DMA measurements on the mechanical response of the dual-cure composite materials, their mechanical behavior was further investigated by means of uniaxial tensile testing on unfilled and fiber-reinforced dumbbell crosslinked specimens. Table 2 summarizes the average values of elastic modulus, maximum tensile strength and elongation at break obtained on all tested samples. During the 3D printing fabrication process, the specimens were exposed to UV light for 15 min.

The elastic modulus of unfilled materials is found to be essentially independent of the amount of photocurable resin in the blend (*E*′ = 2.6 GPa and 2.7 GPa for B33 and B50, respectively), in agreement with the results obtained from DMA analysis. However, by increasing the concentration of photocurable (acrylate) component, both the maximum tensile strength and elongation at break are found to substantially decrease. In particular, values of 35.1 MPa (maximum tensile strength) and 1.8% (elongation at break) are reported for B33 as opposed to 16.0 MPa and 0.6% for B50, thus highlighting a direct correlation between tensile properties of these materials and the amount of thermocurable component (epoxy-based) in the blend. These results suggest that fine control over the tensile properties of these unfilled dual-cure systems can be easily accessed by tuning the chemical composition of the blend, without affecting the stiffness of the resulting 3D printed object. As expected, the addition of reinforcing fibers (both in the case of glass and carbon fibers) allows us to enhance the value of elastic modulus with respect to the unfilled materials (3.5 GPa and 3.9 GPa for B33G5 and B50C5, respectively), with a slightly more efficient reinforcement given by carbon fibers. Interestingly, the maximum tensile strength is found to increase both in GFR (41.7 MPa) and CFR (30.6 MPa) composites compared to the corresponding unfilled counterparts (35.1 MPa and 16.0 MPa for B33 and B50, respectively), thus suggesting an efficient load transfer from the polymer matrix to the fibers made possible by the high level of structural homogeneity in the reinforced materials and the relatively good fiber/matrix interfacial interactions. Similar trends were also found on the elongation at the break.

**Figure 3:**
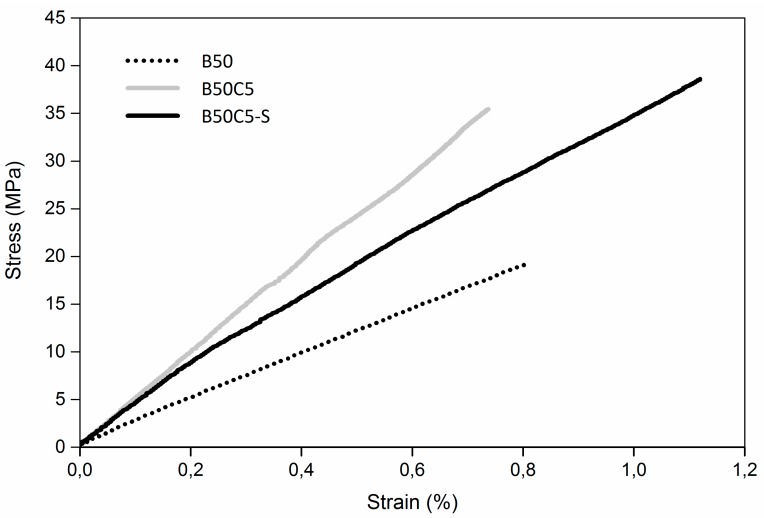
Representative stress–strain curves of UV-3D printed blend (B50) and composites incorporating untreated (B50C5) and sized (B50C5-S) carbon fibers.

In the attempt to further improve the mechanical response of CFR polymer composites, preliminary fiber sizing tests were performed in this work with the aim of enhancing the interfacial adhesion strength between carbon fibers and the polymer matrix. Indeed, it is widely acknowledged that the optimization of the bonding properties of carbon fibers is key to improved mechanical properties of the resulting composite materials [24]. Accordingly, a cryogenic treatment by means of liquid nitrogen [22] was applied on the carbon fibers in order to increase their surface roughness by partially removing the weak thin layer of amorphous carbon present on it, thus leading to the formation of a favorable gripping interface between the reinforcing filler and the matrix. To assess the viability of such treatment and its effect on the mechanical properties of the resulting materials, specimens of CFR polymer composites incorporating sized fibers (B50C5-S) were UV-3D printed and tested by uniaxial tensile measurements. As shown in Table 2 and Figure 3, a synergistic increase of elastic modulus, tensile strength and elongation at break is observed upon fiber sizing, likely originating from the improved fiber/matrix interfacial interactions resulting from the cryogenic sizing treatment. 

### 2.5. Scanning Electron Microscopy (SEM)

**Figure 4:**
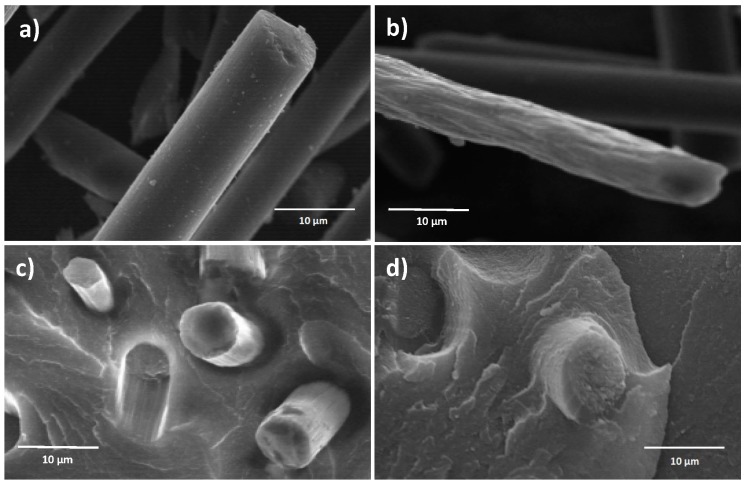
SEM micrographs (2500× magnification) of (**a**) virgin untreated; and (**b**) sized carbon fibers; fracture surface of tensile CFR polymer composites incorporating (**c**) virgin untreated; and (**d**) sized carbon fibers.

To further clarify the effect of sizing on matrix/fiber adhesion, a morphological analysis of the fracture surfaces of CFR composite specimens incorporating untreated and sized fibers was performed by SEM. The results are presented in Figure 4, where also SEM images of the fibers (untreated and sized) are reported, for reference.

The surface of untreated fibers (Figure 4a) appears relatively smooth, with the presence of some small randomly distributed impurities absorbed on it and likely forming during fiber manufacturing. Conversely, the sizing process is found to increase the surface roughness of the fibers as evidenced by the appearance of a higher concentration of longitudinal surface features that may be ascribed to the cryogenic treatment (Figure 4b). In addition, a decrease of fiber diameter is also observed upon sizing. By considering the fracture surface of tensile CFR polymer composite specimens, it appears that upon sizing a stronger bonding between fibers and matrix could be obtained as demonstrated by the presence of a significant amount of residual matrix material around the broken fibers (Figure 4d). Conversely, in CFR specimens incorporating untreated fibers (Figure 4c), partial slipping of the fibers out of the polymer matrix is observed, thus suggesting relatively limited interfacial interaction between the composite components. This evidence correlates well with the results obtained from tensile tests.

### 2.6. UV-3D Printing Tests

In order to demonstrate the printability of the dual-cure IPN composite formulations presented in this work and their potential use in real-life structural applications, different macrostructures were fabricated using a low-cost benchtop UV-3D printing apparatus recently developed in our group (see Figure S3 in the Supplementary Materials) [8].

**Figure 5:**
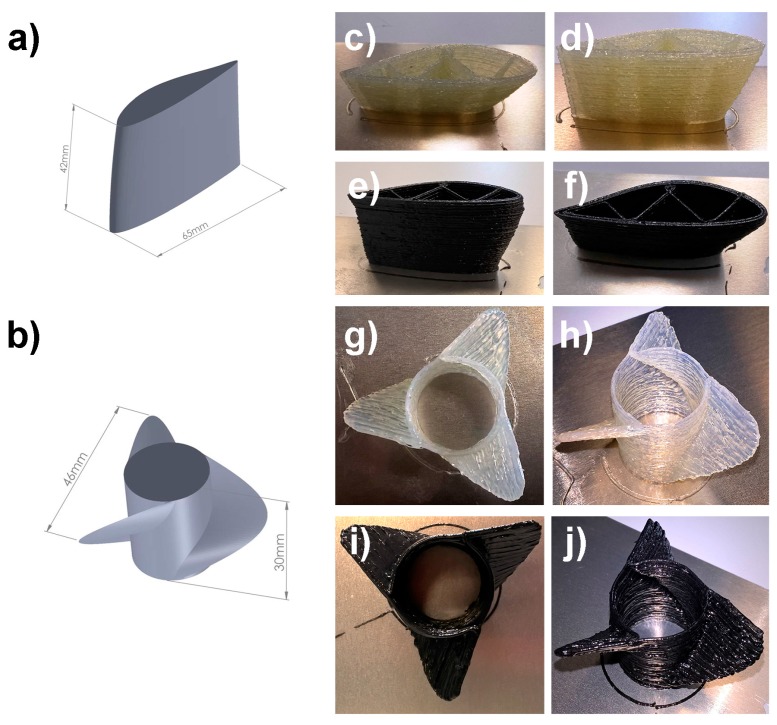
Axonometric projection of the airfoil (**a**) and the propeller (**b**) 3D models used to demonstrate the printability of the GFR and CFR composite formulations developed in this work. UV-3D printed reproduction of the airfoil (**c**–**f**) and the propeller (**g**–**j**) 3D models based on the GFR (**c**,**d**,**g**,**h**) and CFR (**e**,**f**,**i**,**j**) polymer composite formulations developed in this work.

A preliminary printing test was carried out by fabricating a 3D model consisting of vertical column with overhanging features of decreasing angles (varying from 50° at the bottom to 20° at the top) and increasing printing complexity (see Figure S4 in the Supplementary Materials) [16]. In this case, both GFR and CFR composite formulations allowed to achieve excellent reproducibility of the target model (printing speed 10 mm/s, UV-exposure time 30 min), and overhangs up to 20° and 30° could be successfully demonstrated by employing B33G5 and B50C5 formulations, respectively. This represents a first clear indication of the good processability of these fiber-reinforced systems by means of a UV-3D printing apparatus. In addition, the developed fiber-reinforced composite formulations were used as inks to fabricate 3D scale models of complex structural components such as airfoils and propellers (Figure 5, printing speed 5 mm/s, UV-exposure time 30 min and 45 min for airfoils and propellers, respectively). Also in these cases, high-fidelity replicas of the digital model (Figure 5a,b, respectively) could be obtained both when using the GFR and CFR polymer composite formulations developed here. These results demonstrate the possibility of employing the composite formulations developed in this work as highly processable dual-cure inks for UV-3D printing in view of their use as versatile tool for advanced additive manufacturing of structural parts.

It is worth pointing out that the dual-cure mechanism exploited in our work is expected to favour interlayer adhesion between successively-printed layers. Indeed, upon exiting the extrusion nozzle, the partially UV-crosslinked resin filament is deposited on top of another partially UV-crosslinked filament. As a result, partial interpenetration between overlying layers is expected. The final thermal treatment on the UV-assisted 3D printed object allows us to complete the crosslinking process, form the final solid 3D structure and lead to excellent interlayer bonding between successive layers of deposited material.

## 3. Experimental

### 3.1. Materials

The epoxy resin used in this study was bisphenol A diglycidyl ether (BADGE) (Araldite CY 225, Huntsman Advanced Materials (Switzerland) GmbH, Basel, Switzerland), mixed with a hexahydro-4-methylphthalic anhydride (HHMPA, the hardner) and a 2,4,6-tris(dimethylaminomethyl)phenol (DMP-30, the accelerator), both purchased from Sigma Aldrich (Milano, Italy). The photo-curable resin used to obtain the dual-cure blend was an ethoxylated bisphenol A diacrylate (SR349, Sartomer Europe, Colombes, France). Carbon fibers (Panex™ 30 milled Carbon Fibers, 99% carbon, density 1.8 g/mL, fiber diameter 7.2 μm, fiber length 100–150 μm) were kindly provided by Zoltek Carbon Fiber. Glass fibers (FM04, fiber diameter 13 μm, fiber length 200 μm) were kindly provided by Italdry srl. Silica nanoparticles (OX200, average particle size 0.2–0.3 µm) were obtained from Sigma Aldrich.

### 3.2. Preparation of the Composite Inks and 3D Printing

The base thermo-curable component of the blend was prepared by mixing under vigorous stirring BADGE, HHMPA (molar ratio 1:2) and DMP-30 (1 wt. %) at 70 °C for 1 h. To obtain the dual-cure blend, the appropriate concentration of SR349 (33 wt. % for B33, 50 wt. % for B50) was subsequently added to the thermos-curable resin and stirred for 0.5 h at 40 °C. Varying amounts of fumed silica (thixotropic agent) were added to the so-obtained dual-cure formulations by manual stirring for five minutes, to allow for correct 3D printing processability. The final GFR and CFR composite formulations were obtained by adding 5 wt. % of reinforcing fibers to the dual-cure unfilled formulation by manual stirring for five minutes, leading to B33G5 (GFR) and B50C5 (CFR). All prepared inks were 3D printed using a custom-built UV-3D printing apparatus developed in our laboratories, based on an appropriately modified low-cost, home-assembled 3Drag 1.2 benchtop printed (Futura Elettronica, Gallarate, Italy) (see Figure S3 in the Supplementary Materials). A syringe equipped with a 0.84 mm diameter nozzle was used for all printing tests. The two 3 W torches used to provide UV curing have a light emission peaked at 405 nm. The target 3D model was designed by means of Solidworks software (Dassault Systèmes, Vélizy-Villacoublay, France). The resulting .stl file was then processed by means of an open-source slicing software (Cura 2.1.2, Ultimaker B.V., Geldermalsen, The Netherlands) (spiral slicing option, single wall thickness) to obtain the final. Gcode that controlled the movements of the printing apparatus.

The thermal curing cycle after UV-3D-printing was carried out at 140 °C for 120 min in a ventilated oven.

### 3.3. Rheological Characterization of the Composite Inks

The rheological behavior of the blends and the fiber-reinforced composite formulations was investigated using a shear stress-controlled Rheometrics DSR 200 rheometer from 20 to 2000 Pa. All the tests were performed at room temperature. The diameter of the cone-plate geometry mounted on the rheometer was 25 mm with a 0.051 mm gap between them.

### 3.4. Dynamic Mechanical Analysis (DMA)

DMA analyses were performed in three-point bending configuration on a Mettler Toledo DMA/SDTA861 instrument in strain sweep at ambient temperature, and in dynamic scans from 20 °C to 240 °C at 3 °C/min heating rate. The frequency was kept constant (1 Hz).

### 3.5. Differential Scanning Calorimetry (DSC)

DSC analysis was carried out by means of a Mettler Toledo DSC/823^e^ instrument (Mettler Toledo, Novate Milanese, Italy) indium and *n*-hexane calibrated. Samples were heated from 20 °C to 250 °C with a 20 °C/min heating rate in N_2_ environment.

### 3.6. Uniaxial Tensile Tests

Tensile properties were determined at room temperature by means of a Zwick/Roell Z010 (Zwick Roell Italia, Genova, Italy) equipped with a 10 kN load cell and a longstroke extensometer following the standard test method ASTM D638 (Type I specimen, dimensions down-scaled to 60%) for the unfilled dual-cure blend samples. For the GFR and CFR polymer composites, the ASTM D3039/3039M was followed (random-discontinuous fiber orientation, dimensions down-scaled to 60%). All tested specimens were UV-3D printed with a full-shell infill type.

### 3.7. Scanning Electron Microscopy (SEM)

SEM images on carbon fibers and fractured CFR composites were performed with a Carl Zeiss EVO 50 Extended Pressure scanning electron microscope and a Cambridge Stereoscan 360 (Carl Zeiss Italia, Milano, Italy). The acceleration voltage was kept constant at 15 kV. 

## 4. Conclusions

In this work, a new dual-cure polymeric formulation was presented for use as matrix material in GFR and CFR polymer composites for UV-3D printing. The resulting printed macrostructures were found to possess good thermal stability and mechanical properties, as evidenced by DSC and DMA analyses. In addition, uniaxial tensile tests revealed for the first time the efficient reinforcing effect of the fibers on the UV-3D printed objects, both in the case of GFR and CFR systems. Finally, a preliminary study on the use of a sizing treatment on carbon fibers revealed the potential of this approach to improve the mechanical properties of 3D printed CFR parts thanks to the improved fiber/matrix interfacial adhesion. The results of this study give a clear demonstration of the suitability of the developed GFR and CFR composite systems for application in the field of advanced additive manufacturing for structural components.

## Figures and Tables

**Table 1:** Storage modulus *E*′ and *T*_g_ obtained by means of dynamic Mechanical Analysis (DMA) and differential Scanning Calorimetry (DSC) measurements on unfilled and fiber-reinforced (both carbon and glass) blends.

Property	B33	B50	B33G5	B50C5
Storage modulus *E*′ (GPa) ^1^	3.15 ± 1.0	3.15 ± 0.6	4.30 ± 0.3	5.18 ± 0.6
Theoretical prediction of *E*′ (GPa) ^2^	-	-	4.65	5.58
*T*_g,DMA_ (°C)	127	128	155	123
*T*_g,DSC_ (°C)	106	100	105	74

^1^ The reported values of storage modulus were obtained at 25 °C; ^2^ Theoretical prediction as calculated by using the Halpin-Tsai model.

**Table 2:** Average values ^1^ (±standard deviation) of elastic modulus, maximum tensile strength and elongation at break for unfilled (B33 and B50) and fiber-reinforced (B33G5 and B50C5) composite materials. The mechanical properties of CFR composites containing sized carbon fibers are also displayed (B50C5-S).

Property	B33	B50	B33G5	B50C5	B50C5-S
Elastic modulus *E*′ (GPa)	2.6 ± 0.7	2.7 ± 0.4	3.5 ± 0.3	3.9 ± 0.8	4.4 ± 0.9
Maximum tensile strength *σ*_max_ (MPa)	35.1 ± 8.9	16.0 ± 2.9	41.7 ± 5.1	30.6 ± 5.9	33.8 ± 4.9
Elongation at break *ε*_B_ (%)	1.8 ± 0.7	0.6 ± 0.2	1.6 ± 0.1	0.9 ± 0.2	1.0 ± 0.2

^1^ The reported values were obtained by uniaxial tensile tests.

## Supplementary Materials

The following are available online at www.mdpi.com/1996-1944/9/7/583/s1. Table S1: Values of the power-law index *n* and of the consistency index *K* for B33 formulations (dual-cure blend containing 33 wt. % of photocurable acrylic component); Figure S1: Consistency indexes *K* as a function of SiO_2_ concentration for B33 formulations (dual-cure blend containing 33 wt. % of photocurable acrylic component); Table S2: Values of the power-law index *n* and of the consistency index *K* for unfilled (B33 and B50) and CFR (B50C5) composite formulations. For comparison, CFR and GFR composite formulations without the addition of SiO_2_ are also presented (B50C5-noSiO_2_ and B33G5-noSiO_2_, respectively); Figure S2: Consistency indexes *K* as a function of SiO_2_ concentration for unfilled (B33 and B50) and CFR (B50C5) composite formulations. For comparison, CFR and GFR composite formulations without the addition of SiO_2_ are also presented (B50C5-noSiO_2_ and B33G5-noSiO_2_, respectively); Figure S3: Schematic representation of the 3D printer equipped with UV-torches used in this work; Figure S4: 3D digital model (a) and UV-3D printed reproductions of the object used to demonstrate the printability of the GFR (b) and CFR (c) composite formulations developed in this work; Figure S5: 3D slicing model of a representative specimen (obtained using Cura, Ultimaker B.V.) and detail of the UV-3D printing process of a CFR formulation.
