# Supplementary material for: UV-Assisted 3D Printing of Glass and Carbon Fiber-Reinforced Dual-Cure Polymer Composites
Source: Materials (Basel). 2016 Jul 16;9(7):583. doi: 10.3390/ma9070583 (PMC5456860; doi:10.3390/ma9070583)
Supplement: Supplementary file 1 [file materials-09-00583-s001.pdf]

Supplementary Materials: UV-Assisted 3D Printing of Glass and Carbon Fiber-Reinforced Dual-Cure Polymer Composites

Marta Invernizzi, Gabriele Natale, Marinella Levi, Stefano Turri and Gianmarco Griffini

The viscosity curves of all formulations investigated in this work were fitted by the power-law equation (Equation (1) in the main manuscript). The calculated values of the consistency index *K* and power-law index *n* are shown in Tables S1 and S2 and the consistency index *K* is plotted as a function of SiO_2 concentration in the blends in Figures S1 and S2.

Table S1. Values of the power-law index *n* and of the consistency index *K* for B33 formulations (dual-cure blend containing 33 wt. % of photocurable acrylic component).

SiO_2 wt. % in the Blend	*K*	*n*
0 (B33)	22.31	0.99
3	20.56	0.76
5	158.15	0.43
7	795.26	0.11

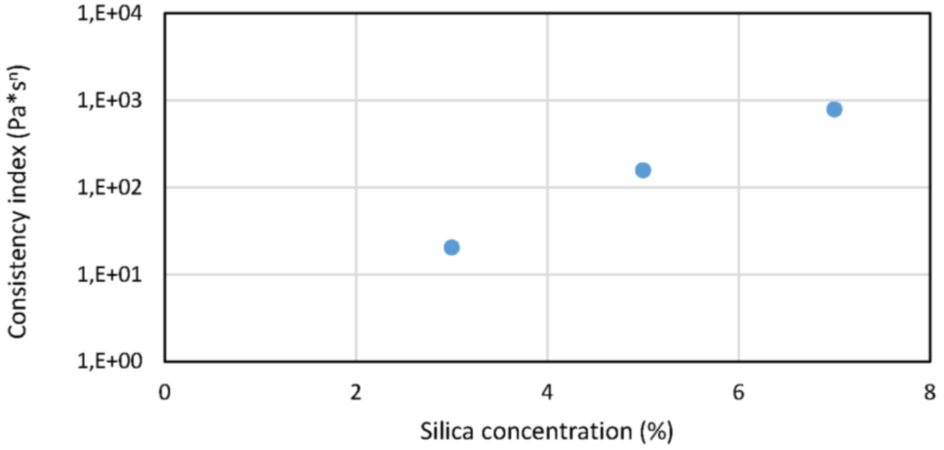

Figure S1. Consistency indexes *K* as a function of SiO_2 concentration for B33 formulations (dual-cure blend containing 33 wt. % of photocurable acrylic component).

Table S2. Values of the power-law index n and of the consistency index *K* for unfilled (B33 and B50) and CFR (B50C5) composite formulations. For comparison, CFR and GFR composite formulations without the addition of SiO_2 are also presented (B50C5-noSiO_2 and B33G5-noSiO_2, respectively).

SiO_2 wt. % in the Blend	*K*	*n*
**B33**	919.16	0.14
**B50**	996.51	0.12
**B50C5**	803.33	0.20
**B33G5-noSiO_2**	4.67	0.97
**B50C5-noSiO_2**	3.46	1.00

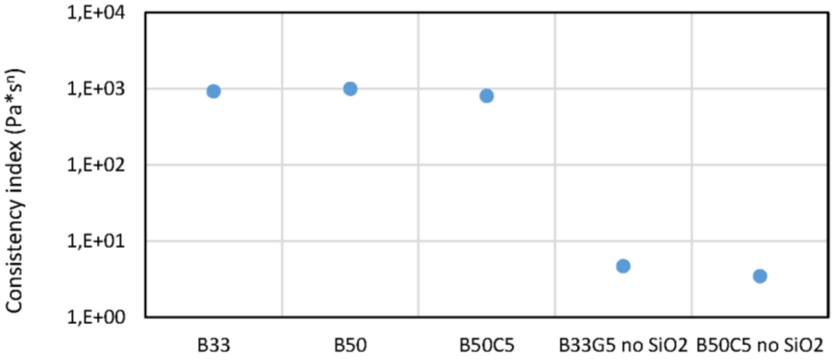

Figure S2. Consistency indexes *K* as a function of SiO_2 concentration for unfilled (B33 and B50) and CFR (B50C5) composite formulations. For comparison, CFR and GFR composite formulations without the addition of SiO_2 are also presented (B50C5-noSiO_2 and B33G5-noSiO_2, respectively).

A low-cost home-assembled 3Drag 1.2 benchtop printer (Futura Elettronica, Italy) incorporating a syringe dispenser with a 0.84 mm diameter nozzle was equipped with two 3W UV-A torches (WF-501B by Ultrafire Ltd., Shenzhen, China) on the printing head with light emission peaked at 405 nm (Figure S3).

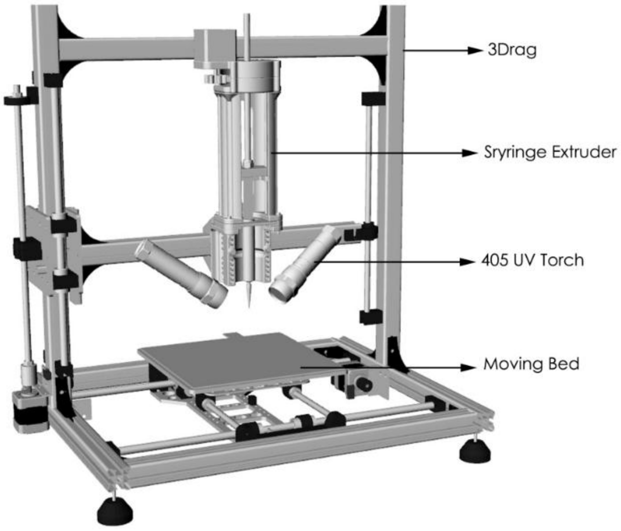

Figure S3. Schematic representation of the 3D printer equipped with UV-torches used in this work.

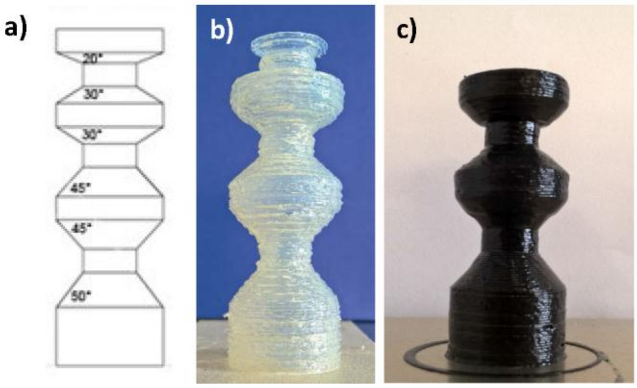

Figure S4. 3D digital model (**a**) and UV-3D printed reproductions of the object used to demonstrate the printability of the GFR (**b**) and CFR (**c**) composite formulations developed in this work.

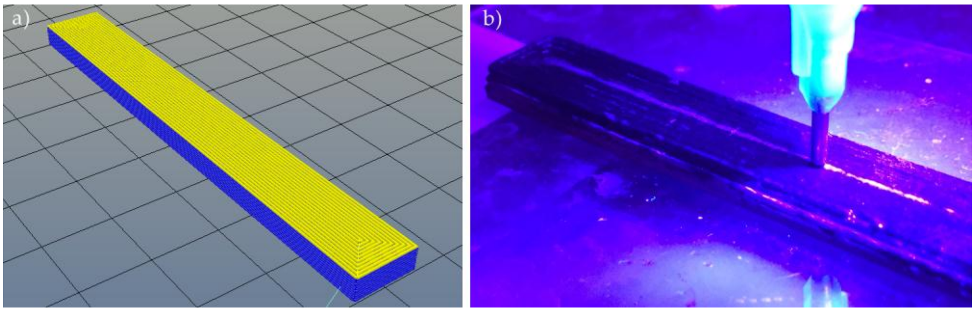

Figure S5. (**a**) 3D slicing model of a representative specimen (obtained using Cura, Ultimaker B.V.) and (**b**) detail of the UV-3D printing process of a CFR formulation.
